# Guest Editorial: From neuroscience to neuro-rehabilitation: transferring basic neuroscientific principles from laboratory to bedside

**DOI:** 10.1186/1743-0003-10-6

**Published:** 2013-01-21

**Authors:** Alexander Koenig, Andreas Luft, Iahn Cajigas

**Affiliations:** 1 Harvard Medical School, Spaulding Rehabilitation Hospital, Motion Analysis Lab, Boston, MA, USA; 2 University Hospital Zurich, University of Zurich, Zurich, Switzerland; 3 Harvard/MIT Division of Science and Technology and Harvard Medical School, Boston, MA, USA

## Abstract

Several new approaches for treatment of Central Nervous System (CNS) disorders are currently under investigation, including the use of rehabilitation training strategies, which are often combined with electrical and/or pharmacological modulation of spinal locomotor circuitries. While these approaches show great promise in the laboratory setting, there still exists a large gap in knowledge on how to transfer these treatments to daily clinical use. This thematic series presents a cross section of cutting edge approaches with the goal of transferring basic neuroscience principles from the laboratory to the proverbial "bedside".

## Editorial

Central nervous system (CNS) disorders such as stroke, spinal cord injury (SCI), traumatic brain injury, multiple sclerosis or Parkinson’s cause a variety of functional impairments ranging from impaired control of gait or reach through to deficits in cognition and autonomic dysfunction [1]. Development of successful treatment strategies that aim to restore a patient’s quality of life require a detailed understanding of the underlying pathophysiology of injury/disease together with a holistic view of how the impairment affects an individuals’ daily life.

Based on simultaneous exploration of animal models and clinical trials on humans, the last decades brought significant improvements in our knowledge of pathophysiology of numerous neurological disorders. From improved understanding of the basic neuroscience and biochemistry associated with CNS damage and recovery from it, many new experimental therapeutic approaches have emerged, including: neural regeneration [2], modulation of spinal excitability via pharmacological or stimulation-based approaches [3,4] and functional rehabilitation-training strategies [5]. While our understanding of neural plasticity and repair continues to grow, these approaches are not likely to reach the clinical maturity over this decade. Recent clinical trials such as the Geron Trials, (ClinicalTrials.gov number NCT01217008) on the use of neuro-regenerative drugs were halted due to financial reasons.

Focusing on the CNS integrated control of movement, there are several approaches that have been successful in the laboratory setting, including the use of rehabilitation training strategies, which are often combined with electrical and/or pharmacological modulation of spinal locomotor circuitries to improve functional outcomes in people living with CNS impairment. To this end, this thematic series presents some of the current work that bridges the gap from basic science to clinical application. The articles represent a cross section of cutting edge approaches with the goal of transferring basic neuroscience principles from the laboratory to the proverbial “bedside”. The contributions explore the feasibility and efficacy of novel activity-dependent training strategies for both upper and lower extremities and the development of new technologies for spinal stimulation.

Hubli and Dietz set the stage by reviewing the physiological basis and state of the art of rehabilitation after spinal cord injury. The authors emphasize the importance of task specific sensory clues, which were shown to be highly relevant for neural plasticity and consecutive optimization of functional recovery. As already shown in animal experiments [4], they conclude that, in the future, high-intensity movement training combined with next generation spinal stimulators and neuro-modulatory drug interventions could facilitate appropriate neuroplasticity, and thereby improve rehabilitation efficacy in humans.

Starting with healthy subjects, Zimmermann et al. present a new brain-computer-interface approach for rehabilitation paradigms involving the upper extremities. The authors take a first step towards real-time control of rehabilitation robots or prosthetics based online decoding of movement intention during a finger-pinching task. Using functional Near-InfraRed-Spectroscopy (fNIRS) recordings from motor-cortex M1 of healthy subjects, in combination with physiological signals (including heart rate and skin conductance), they detect movement onset in real time. By decoding the motor intention in real-time and relaying it to a robotic device that generates the desired movement, the resulting sensory signals better match the initial motor intention and command. In current rehabilitation paradigms, particularly in severely affected patients, the physical therapist generates functionally relevant sensory signals to the CNS by moving the patient’s limbs; these sensory signals are not necessarily temporally congruent with the movement intention generated by the patient. The faster setup time of fNIRS compared to other brain computer interfaces, such as EEG, promises easier clinical use. While it remains to be confirmed that patients with motor cortex lesions would be able to activate the respective adjacent preserved or undamaged motor cortex regions to be able to use this brain computer interface, this work opens a whole line of research on novel treatment paradigms.

Koopmann et al. take on the problem of designing subject-specific lower extremity robotic rehabilitation interventions after stroke. While rehabilitation robots in the past followed a “one fits all” approach, the authors propose to divide the gait cycle into sub-tasks such as weight support, forward propulsion or control of the swing phase. Focusing on swing phase in this paper, the authors developed an assist-as-needed controller for the LOPES rehabilitation robot [6] that can reconstruct an impaired individual’s gait pattern and modulates step height gradually from their current baseline to a more desirable pre-injury trajectory. The underlying hypothesis is that a rehabilitation protocol tailored to an individuals’ specific gait impairment should lead to greater functional improvement. In twelve healthy subjects and six stroke patients, the authors demonstrate that modulation of step height during the swing phase can be iteratively modulated. By using catch trials during which the modulating force applied to the limbs is abolished for only one step, they show that subjects and patients learn the new step height and do not just rely on the robotic support. In the future, a set of such controllers, each able to modulate a specific subtask, could be used to personalize (tailor) the robotic intervention paradigms to each person's individual needs and requirements.

Also on the topic of individualized therapy interventions, Gad et al. present their work on designing and evaluating a new high-density array for spinal stimulation in rats with traumatic spinal cord injury. While most current implantable stimulators rely on broad tonic stimulation of spinal segments, the authors hypothesize that high density arrays allowing high spatial resolution stimulation of select motor circuitry will allow training of specific gait related functions such as ankle or knee flexion during swing or the amount of weight bearing during stance. In addition, such arrays will allow addressing the problem of potential spontaneous movement (drift) of the stimulating electrodes within the spinal tissue. Showcased in a single case study on one rat, the authors provide evidence that the stimulation site is of crucial importance to elicit stepping in the rat. In addition, the same device can be used to assess functional conductivity between specific motor pools via motor-evoked potentials, and could thereby be used as a diagnostic and rehabilitation training tool. As the optimal location for stimulation can vary from animal to animal, from patient to patient and even from day-to-day, the improved spatial resolution of such arrays could in the future be expected to allow tuning the stimulator to a pattern optimally adjusted to each patient’s individual motor deficit.

Finally, Patten et al. investigate the efficacy of a novel training paradigm, namely high intensity, dynamic resistance power training, on clinical outcome scores in 19 stroke survivors. In a cross-over design, the authors show that a dose equivalent combination of functional training and strength training yields larger functional improvements that are better retained at six months post-intervention. This supports the hypothesis that intensity of movement therapy is crucial for maximal recovery after neurological injury.

Taken together, these articles highlight the importance of patient-centered approaches towards improving functional outcomes and quality of life. All these approaches look at specific aspects of the rehabilitation continuum. As these techniques mature into clinical reality, they provide a path forward for individualized rehabilitation programs, consisting of modules specifically customized to address a patient’s underlying functional deficits. Given the heterogeneity of deficits observed clinically, an adaptable combination of interventions will likely be required to address each individual’ impairments and needs. Determining the appropriate "dose response" and the best combination of approaches remains to be scientifically and clinically validated.
